# Disease Duration Influences Gene Expression in Neuromelanin-Positive Cells From Parkinson’s Disease Patients

**DOI:** 10.3389/fnmol.2021.763777

**Published:** 2021-11-11

**Authors:** Katarína Tiklová, Linda Gillberg, Nikolaos Volakakis, Hilda Lundén-Miguel, Lina Dahl, Geidy E. Serrano, Charles H. Adler, Thomas G. Beach, Thomas Perlmann

**Affiliations:** ^1^Department of Cell and Molecular Biology, Karolinska Institutet, Stockholm, Sweden; ^2^Ludwig Institute for Cancer Research, Stockholm, Sweden; ^3^Banner Sun Health Research Institute, Sun City, AZ, United States; ^4^Department of Neurology, Mayo Clinic College of Medicine, Mayo Clinic Arizona, Scottsdale, AZ, United States

**Keywords:** Parkinson’s disease, gene expression, RNA sequencing, disease duration, neurodegenerative disease, cell death, neuroprotection

## Abstract

Analyses of gene expression in cells affected by neurodegenerative disease can provide important insights into disease mechanisms and relevant stress response pathways. Major symptoms in Parkinson’s disease (PD) are caused by the degeneration of midbrain dopamine (mDA) neurons within the substantia nigra. Here we isolated neuromelanin-positive dopamine neurons by laser capture microdissection from *post-mortem* human substantia nigra samples recovered at both early and advanced stages of PD. Neuromelanin-positive cells were also isolated from individuals with incidental Lewy body disease (ILBD) and from aged-matched controls. Isolated mDA neurons were subjected to genome-wide gene expression analysis by mRNA sequencing. The analysis identified hundreds of dysregulated genes in PD. Results showed that mostly non-overlapping genes were differentially expressed in ILBD, subjects who were early after diagnosis (less than five years) and those autopsied at more advanced stages of disease (over five years since diagnosis). The identity of differentially expressed genes suggested that more resilient, stably surviving DA neurons were enriched in samples from advanced stages of disease, either as a consequence of positive selection of a less vulnerable long-term surviving mDA neuron subtype or due to up-regulation of neuroprotective gene products.

## Introduction

Parkinson’s disease (PD) is the most common neurodegenerative motor disorder, affecting 1−2% of individuals over 65 years. A hallmark of the disease is the progressive degeneration of midbrain dopamine (mDA) neurons in the substantia nigra pars compacta (SNc) and the accumulation of intraneuronal Lewy bodies containing misfolded α-synuclein in brainstem and neocortex (Damier et al., 1999). In particular during early stages of disease, motor functions are affected and major symptoms include bradykinesia, rigidity, tremor and postural instability. Parkinson’s disease patients also suffer from non-motor symptoms such as depression, olfactory dysfunction, sleep disorders, hallucinations and cognitive impairments (Chaudhuri and Schapira, 2009). Other diseases are also associated with Lewy bodies, including dementia with Lewy bodies. In addition, Lewy bodies are often found in aged brains at autopsy also from individuals lacking symptoms. Such cases are often referred to as incidental Lewy body disease (ILBD). It remains unknown if ILBD represents a pre-clinical stage PD or if it is a unique pathological abnormality but as ILBD has been shown to have nigrostriatal dopaminergic losses midway between normal and PD subjects, it remains a likely candidate for a preclinical PD stage (Gibb and Lees, 1988; Beach et al., 2008; DelleDonne et al., 2008; Dickson et al., 2008; Adler et al., 2010; Iacono et al., 2015).

Currently used PD treatments alleviate motor dysfunction by mDA signaling compensation, usually by administration of levodopa or other drugs that increase dopamine signaling. However, side effects develop over time and the treatment usually becomes progressively ineffective. Importantly, treatments increasing dopamine signaling does not slow neurodegeneration. A better understanding of the neurodegenerative mechanisms underlying the disease may result in identification of new molecular targets and permit the development of more effective therapies (Armstrong and Okun, 2020).

Comprehensive histopathological characterization of postmortem tissue of a large sample of PD cases of different disease duration suggests that both the soma and the axon of mDA neurons is affected early after diagnosis, with loss of mDA neuron markers both in the SNc and in the striatum. Degeneration of mDA neurons occurs rapidly within the first 4 years post diagnosis and seems to progress rather slowly after the initial cellular decline (Kordower et al., 2013). Moreover, despite the *fact* that idiopathic PD has a rather limited genetic component, studies of familial PD-linked genes have resulted in significant advances in the understanding of the molecular pathways implicated in PD. Thus, SN mDA neuron pathology has been associated with mitochondrial dysfunction, oxidative stress, excitotoxicity, inflammation and abnormal protein handling and folding (Johnson et al., 2019).

In addition to histopathological and genetic analyses, another tool that can provide insights into the pathology of PD is the analysis of changes in global gene expression patterns in samples from *post-mortem* PD brains. Gene expression profiling experiments have previously been performed on mRNA extracted from homogenates of the SN. Since mDA neurons only constitute a subpopulation of neurons and glial cells within the dissected SN such samples contain relatively few mDA neurons in relation to other cell types. Moreover, *post-mortem* samples are usually derived after prolonged disease when only few mDA neurons remain (Kordower et al., 2013). Thus, mDA neurons are severely underrepresented in such samples. To avoid influence from other cell types in the analysis, mDA neurons can be isolated directly by laser-capture microdissection (LCM). Several previous studies have used this or other methods to isolate mRNA from the SN and used mRNA for analyses in hybridization chip arrays for detection of genome-wide gene expression (Cantuti-Castelvetri et al., 2007; Bossers et al., 2009; Simunovic et al., 2009; Zheng et al., 2010; Elstner et al., 2011; Riley et al., 2014). In another study, LCM was used to specifically quantify the expression of *SNCA* (α-synuclein) mRNA (Gründemann et al., 2008). However, direct measurement mRNA expression by means of massively parallel RNA sequencing (RNAseq) offers a more sensitive analysis, improved dynamic range and with less technical variability as compared to different chip array platforms (Nichterwitz et al., 2016).

Tissue sampled from PD patients in previous studies were usually diagnosed many years before tissue collection and reports on how gene expression is affected during disease progression is lacking. Importantly, histopathological analysis of disease duration has demonstrated a profound loss of mDA neurons in the SN during the first years after diagnosis with nearly negligible loss thereafter (Kordower et al., 2013). Thus, it can be assumed that previous studies have mostly analyzed gene expression in mDA neurons that are stably maintained after the initial rapid phase of mDA neuron decline.

In this study, we used RNAseq to analyze genome-wide gene expression in mDA neurons isolated by LCM from postmortem cases of PD. Patients’ samples are derived both early after diagnosis and after prolonged disease duration. Cases of ILBD and age-matched controls were also included in the analysis. We observed distinct transcriptional profiles corresponding to the differential duration of PD and we show that the transcriptional changes in ILBD are distinct from those seen in PD.

## Materials and Methods

### Samples

Fresh snap-frozen human *post-mortem* tissue was provided by the Banner Sun Health Research Institute Brain and Body Donation Program of Sun City, Arizona, United States, where all human subjects donate after signed informed written consent [Western Institutional Review Committee (WIRB), Seattle, Washington] (Beach et al., 2015). Sample data used in the preparation of this article were obtained from the Arizona Study of Aging and Neurodegenerative Disorders (AZSAND) database.^1^ Detailed information about the samples analyzed is provided in Supplementary Figure 1. Controls (*n* = 10) didn’t have any neurodegenerative disease and were selected to match the age and the gender of the PD cases. Since loss of dopaminergic markers occurs rapidly and is completed by 4 years post-diagnosis (Kordower et al., 2013), we selected two groups of PD cases: early post-diagnosis 2-4 years (*n* = 6) and late post-diagnosis 5-24 years (*n* = 6). ILBD cases (*n* = 7) were also included. Only samples with a *post-mortem* index < 5h were included. To get robust results, we used ≥six cases per condition (Schurch et al., 2016).

### Laser Capture Microdissection

Cryosectioning was used to obtain 10 μm thick sections of the ventral midbrain, which were mounted on membrane glass slides (Zeiss, 415190-9041-000) that were kept at −20∘C during sectioning and subsequently stored at −80∘C. Prior to LCM, the tissue was dehydrated by a series of ethanol washes (75% 30 s, 95% 30 s, 100% 30 s). The Leica LMD7000 system was used to capture NM+ neurons from the SNc. 10 controls, 12 PD and 7 individuals with ILBD were analyzed. Cells were cut at ×40 magnification with a minimum laser power and collected into lysis buffer (Picelli et al., 2013). From each SNc, 4–12 pooled samples of 100–200 micro-dissected cells were collected. As a reference, 18 samples were also collected from the cortex of control brains to provide a non-DA source of RNA. Cortex neurons were visualized using a quick Histogene (Arcturus) staining.

### cDNA Library Preparation and Sequencing

Libraries for RNA-seq were generated using the Smartseq2 protocol (Picelli et al., 2013). cDNA libraries were tagmented using home-made Tn5 enzyme (Picelli et al., 2014) and Nextera dual indexes (Illumina). The cDNA quality was checked on high-sensitivity DNA chip (Agilent Bioanalyzer). Illumina HiSeq 2000 was used for sequencing, giving 43 base pair reads after de-multiplexing.

### Read Alignment and Quality Control

Following de-multiplexing, reads were aligned to the hg19 human genome using STAR v2.3.0 and filtered for uniquely mapped reads (Dobin et al., 2013). Gene expression was calculated as reads per kilobase gene model and million mappable reads (RPKMs) for each transcript in Ensembl release 69 using rpkmforgenes (Ramsköld et al., 2009). The low-quality libraries were filtered out based on following criterias: <10.3% uniquely mapping reads, >68% fraction mismatches, <10.5% exon mapping reads, >57% 3′mapping, < 26% of all genes detected, <100000 normalization reads. 270 samples passed the quality control.

### Gene Expression and Pathway Analysis

Principle Component Analysis (PCA) was used to analyze how individual samples relate to each other on overall gene expression profiles and DESeq2 package (Love et al., 2014) to determine the differentially expressed genes between sets of samples.

Pathway enrichment analysis was performed using the Toppgene suite (Chen et al., 2009) on differentially expressed genes detected with DESeq2 that fulfilled the following criteria: padj < 0.05, mean RPKM value in control samples > 1 and 1.5 fold change.

## Results

### Tissue Preparation, Neuron Collection and Library Preparation

**FIGURE 1:**
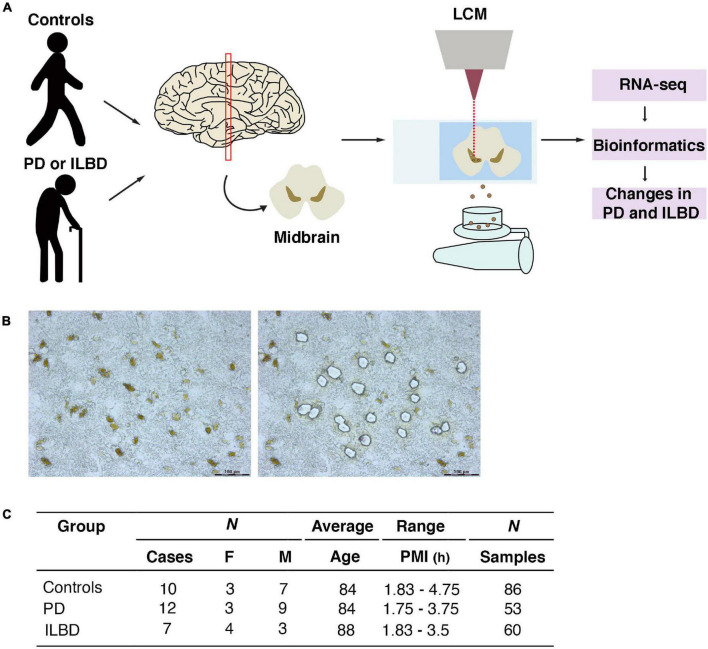
LCM and RNA-seq analysis of mDA neurons from postmortem cases of Parkinson’s disease, incidental Lewy body disease and age-matched controls. **(A)** Schematic overview of experimental design. Midbrain sections were used to isolate neuromelanin positive DA neurons by LCM and processed for RNA-seq. **(B)** Micrographs of ventral midbrain neuromelanin positive cells before and after LCM. **(C)** Parkinson’s disease, incidental Lewy body disease and control samples analyzed in the study.

Snap-frozen tissue blocks, received from the Banner Sun Health Research Institute Brain and Body donation program, were used to obtain cryo-sections of the ventral midbrain. Laser capture microdissection (LCM) was used to isolate mDA neurons from postmortem cases of sporadic PD, ILBD and age-matched controls (Figure 1A and Supplementary Figure 1). mDA neurons were identified by the presence of neuromelanin (NM) and dissected using LCM (Figure 1B). Several replicates of 100–200 individually isolated mDA neurons per individual (either PD, ILBD or age-matched controls) were collected. Cortical neurons from control brains (*n* = 18 pools), visualized by Histogene quick staining, were also collected by LCM in order to provide a reference source of mRNA from a brain region devoid of mDA neurons. Libraries for RNA sequencing were generated by the Smart-seq2 protocol (Picelli et al., 2013). Figure 1C summarizes cases used in the study and the number of analyzed samples per case, while Supplementary Figure 1 provides more information on all cases included in the study.

**FIGURE 2:**
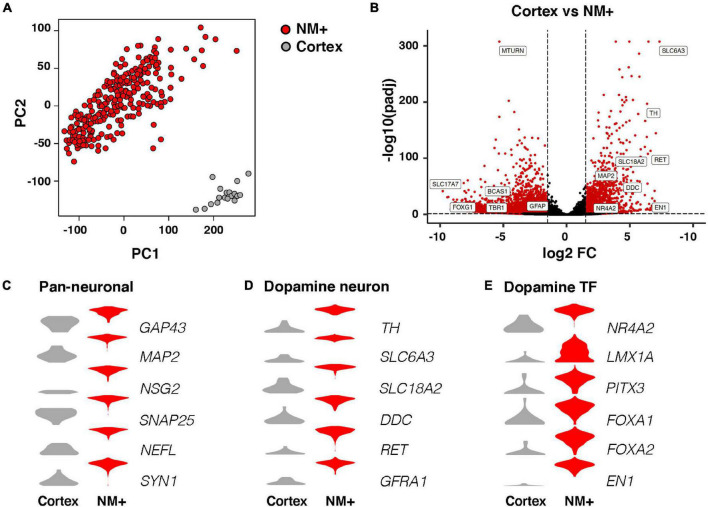
RNA-seq analysis of neuromelanin positive cells in comparison to cortex. **(A)** Two dimensional PCA (PC1, PC2) of NM+ neurons (252 samples) and cortical neurons (18 samples). Samples are color-coded by cell type. **(B)** Volcano plot showing fold changes of individual genes in the DESeq2 analysis. Red: higher expression in NM+ samples (right side of the plot) or cortex samples (left side of the plot), (padj < 0.05, RPKM > 1, fold change > 1.5), Black: no significant difference. **(C–E)** Expression level per cell type for indicated genes in log_2_(RPKM+1) in y-axes. Genes represent markers for pan-neuronal cells **(C)**, dopamine neuron **(D)**, and dopamine transcription factors **(E)**.

Libraries were sequenced (see Methods for details) and analyzed by various bioinformatic methods. The Spearman coefficient between biological replicates of neuromelanin-positive (NM+) cell samples averaged 0.78 and was not below 0.57 for any sample, revealing a very high level of overall correlation (Supplementary Figure 2A). Principal component analysis (PCA) based on all variable genes showed that NM+ samples from healthy controls clustered separately from cortex samples along the second principal component (PC2) (Figure 2A). Further comparison of cortex and NM+ samples revealed that 8845 genes were differentially expressed in the two populations (padj < 0.05, RPKM in controls > 1, fold change > 1.5; Figure 2B and Supplementary Table 1). Known dopaminergic markers such as *TH, SLC6A3, EN1* and *PITX3* were identified among top-ranked genes enriched in NM+ samples demonstrating that these samples provide a good representation of the transcriptome of human mDA neurons (Figures 2B–E). Moreover, the comparison demonstrated that pan-neuronal markers such as *GAP43* and *MAP2* were enriched in the NM+ cells (Figure 2C) while glial cell markers were identified in cortex samples where dissection did not discriminate between neurons and other cell types including astrocytes and oligodendrocytes (Supplementary Figure 2B). This difference was also apparent from gene ontology analysis (Supplementary Figure 2C).

### Differential Gene Expression in Parkinson’s Disease Samples

**FIGURE 3:**
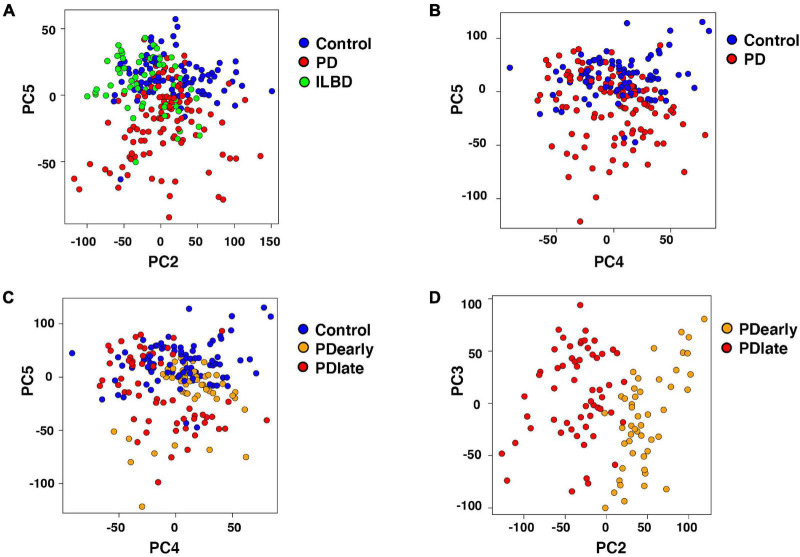
Segregation of Parkinson’s disease samples influenced by disease progression. **(A)** Two dimensional PCA (PC2, PC5) of Control, Parkinson’s disease and incidental Lewy body disease samples. PC5 separates Parkinson’s disease from Control and incidental Lewy body disease samples. Samples are color-coded by disease. **(B,C)** Two dimensional PCA (PC4, PC5) of Control and Parkinson’s disease samples. Samples are color-coded by disease **(B)** and disease duration **(C)**. PC5 separates Parkinson’s disease from Control samples. **(D)** Two dimensional PCA (PC2, PC3) of PDearly and PDlate samples. PC2 separates PDlate from PDearly samples. Samples are color-coded by disease duration.

Interestingly, samples visualized by PCA showed that ILBD and control samples clustered mostly together while a large proportion of PD samples clustered separately along one of the principle components (PC5; Figures 3A,B). Along other PCs, separation was not evident (Supplementary Figures 3A,B). Thus, the analysis detected gene expression profiles that differed in PD samples as compared to controls and ILBD samples. In addition, disease duration affected the transcriptional profile of NM+ cells from PD samples along PC2 as evident from different clustering when comparing samples derived from patients with a disease duration of 2-4 years (PDearly) with those with disease duration of 5 to 24 years (PDlate); (Figures 3C,D). We next considered how additional parameters affected the transcriptional profile of PD samples and found that samples did not cluster separately across any principal component based on either gender, post-mortem intervals (PMI; < 2.5 h versus > 2.5 h) or patient age (< 80 years versus > 80 years) (not shown); however, consistent with the observed different clustering of PDearly vs. PDlate samples, a trend for separation along PC2 was also seen when comparing Braak stages (I, II versus III, IV; Supplementary Figure 3E). Thus, disease duration appears to have an influence on transcriptional profiles in mDA neurons remaining in PD patients at the time of death.

**FIGURE 4:**
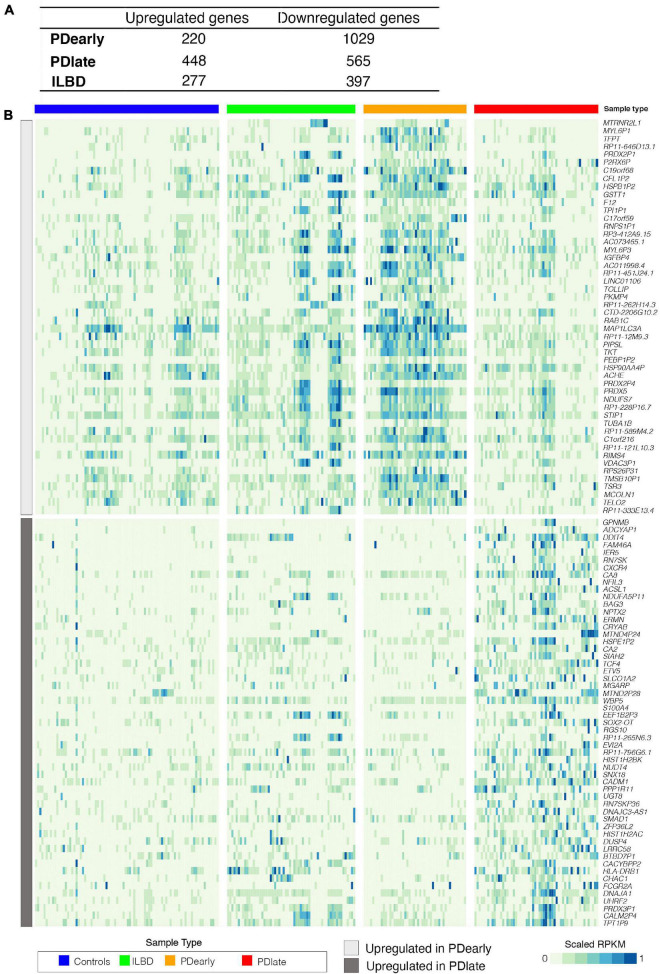
Gene expression changes in PDearly, PDlate and incidental Lewy body disease. **(A)** Number of up- and down-regulated genes in PDearly, PDlate and incidental Lewy body disease (padj < 0.05, RPKM > 1, fold change > 1.5). **(B)** Heatmap visualizing expression of top-upregulated genes in PDearly and PDlate. Sample types are indicated by color coding. Genes upregulated early are in the top part of the heatmap (light gray) while genes upregulated late are in the bottom part (dark gray).

Consistent with PCA demonstrating differences between control and PD samples, DESeq2 statistical analysis (Love et al., 2014) identified hundreds of differentially expressed genes. In PDearly samples, 220 genes were expressed at a higher level and 1029 genes were expressed at a lower level compared to healthy controls (Supplementary Table 2); in PDlate samples, 448 genes were more highly expressed and 565 genes were expressed at a lower level (Supplementary Table 3); in ILBD samples, 277 genes were more highly expressed and 397 genes were expressed at a lower level (Supplementary Table 4), (padj < 0.05, RPKM in controls > 1, fold change > 1.5; Figure 4A). Figure 4B displays a heat map visualizing selected top enriched genes in NM+ neurons from PDearly (orange) and PDlate (red) and Supplementary Figure 4B from ILBD. Top downregulated genes are shown in Supplementary Figure 5.

We focused on the top 100 up- or down-regulated genes (padj < 0.05, RPKM in controls > 1, fold change > 1.5) in PDearly, PDlate and ILBD samples and searched literature in order to identify genes that have been previously associated to PD. A striking observation in PDlate samples was the higher expression of several genes shown previously to confer neuroprotection, such as the pituitary adenylate cyclase activating polypeptide *ADCYAP1* (Reglõdi et al., 2004) the calcium-binding protein *S100A4* (Pedersen et al., 2004), the transcription factor *NFIL3* (Tamai et al., 2014), the regulator of G-protein signaling *RGS10* (Tansey and Goldberg, 2010). Neuroprotective gene such as *PRDX5* (Plaisant et al., 2003) was also expressed at a higher level in PDearly cases suggesting that some neuroprotective mechanisms are quite active also during early stages of the disease. Genes previously shown to be more highly expressed in SNc from PD patients, including *DDIT4* (Malagelada et al., 2006) and *NPTX2* (Moran et al., 2008), were also found to be enriched in PDlate cases. Of interest is also that variants of several regulated genes in both PDearly and PDlate samples, including *GSTT1* (Wang et al., 2014), *CXCR4* (Bonham et al., 2018), *BAG3* (Cao et al., 2017) and *HLA-DRB1* (Hollenbach et al., 2019), were previously associated with PD.

Relatively few genes expressed at lower levels in PD samples were found to be associated with PD. However, *LGALS3* (Galectin-3), a gene linked to neuroinflammation, was expressed at a lower level in PDearly samples and a BCL2 antagonist, was also expressed at lower levels in PDearly samples [see e.g., (Bernardini et al., 2019)]. BAK1, the gene encoding a presumptive target of the E3 ubiquitin ligase Parkin, was expressed at lower levels in PDlate samples. Notably, quite many genes behaved in an opposite manner being enriched in PDearly samples but expressed at lower levels in PDlate samples. This further underscores the different profiles of gene expression seen in samples derived from patients at either early or late stages of disease (Supplementary Tables 2, 3).

**FIGURE 5:**
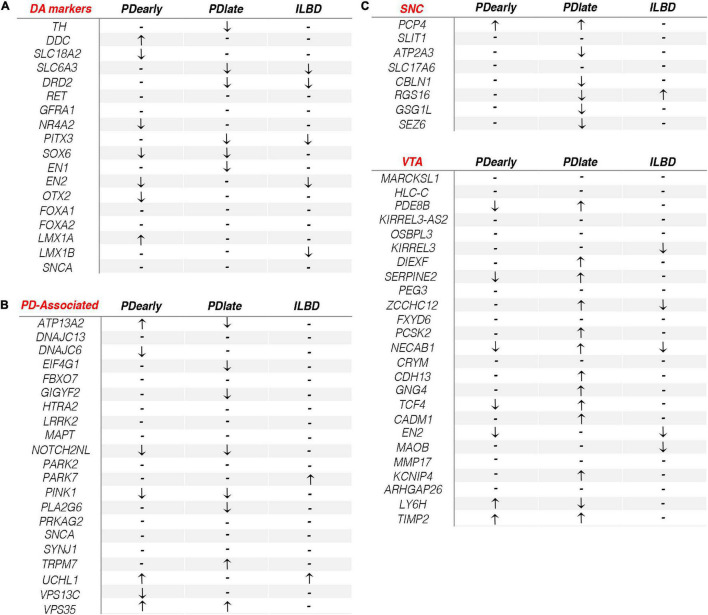
Dopamine related gene expression changes in PDearly, PDlate and incidental Lewy body disease. **(A–C)** Arrows indicate significant (padj < 0.05, RPKM in controls > 1) up- or down-regulation of typical DA markers **(A)**, PD-associated genes **(B)** and SNC/VTA genes **(C)** in PDearly, PDlate and incidental Lewy body disease samples. Hyphen indicates nonsignificant change.

We next focused on known mDA neuron markers and genes that cause familial PD (padj < 0.05, RPKM in controls > 1). The expression of known mDA neuron markers *RET*, *GFRA1*, *LMX1B*, and *FOXA2* were not significantly affected in either PD group. Expression of *DDC* and *LMX1A* were the only genes that were significantly increased in PDearly but not in PDlate samples (Figure 5A). *SLC18A2*, *NR4A2, EN2*, and *OTX2* were expressed at lower level only in PDearly, while *TH*, *SLC6A3*, *DRD2*, *EN1*, and *PITX3* were low only in PDlate samples (Figure 5A). *SOX6*, encoding a transcription factor specifically expressed in SNc but not in ventral tegmental area (VTA), was previously shown to be expressed at lower levels in NM+ cells in PD patients (Panman et al., 2014). Consistent with this result *SOX6* was the only analyzed mDA neuron marker that was significantly expressed at lower level in both PDearly and PDlate cases (Figure 5A). Most genes causing familial forms of PD did not show differential expression in any PD cohort as compared to controls. *UCHL1* was significantly enriched only in PDearly cases, *ATP13A2* was enriched in PDearly but lower than controls in PDlate cases, while *GIGYF2*, *EIF4G1*, and *PLA2G6* were lower in PDlate but not in PDearly cases. *PINK1* and *NOTCH2NL* were the only familial PD-associated genes that was expressed at lower levels in both early and late PD cases (Figure 5B).

The diversity of mDA neurons within the ventral midbrain is substantial. In PD, mDA neurons of the ventral-lateral SNc are more vulnerable than mDA neurons localized at a dorsal-medial region of the SNc and within the VTA (Gibb and Lees, 1991). Although tissue samples were dissected from the substantia nigra it is likely that NM+ cells isolated by LCM in this study represents mDA neurons belonging to different subtypes and with variable vulnerability (Poulin et al., 2020). A recent study identified markers specific for human SNc and VTA mDA neurons, respectively (bioRxiv 334417; doi: https://doi.org/10.1101/334417). The study defined 33 markers that can distinguish between human VTA and SNc mDA neurons. We assessed if any of these genes were enriched in either PDearly, PDlate or incidental Lewy body disease. Interestingly, a clear trend was observed when comparing the different samples (Figure 5C). Accordingly, the majority of VTA markers were significantly enriched in PDlate samples but not in PDearly samples indicating that surviving cells isolated from PDlate samples are enriched for cells with VTA or “VTA-like” gene expression profiles. In contrast, although loss of neurons within the SNc has been reported in ILBD (40%), VTA markers were not enriched in ILBD samples (Iacono et al., 2015) (Figure 5C).

### Overlap of Differential Gene Expression in Parkinson’s Disease and Incidental Lewy Body Disease Samples

The overlap between differentially expressed genes in PD and ILBD samples was limited (Supplementary Figure 4A). Of note, among genes that are differentially expressed in both PD and ILBD samples, several are among those that previously have been associated with PD. Thus, *GPNMB*, *PRDX5*, *GSTT1*, *HLA-DRB1*, and *DDIT4* were enriched in both ILBD and PD samples and linked to PD as described above. Moreover, *LGALS3*, a gene associated with neuroinflammation, was expressed at reduced levels in both PD and ILBD samples.

### Gene Ontology Terms Enriched in Parkinson’s Disease and Incidental Lewy Body Disease

**FIGURE 6:**
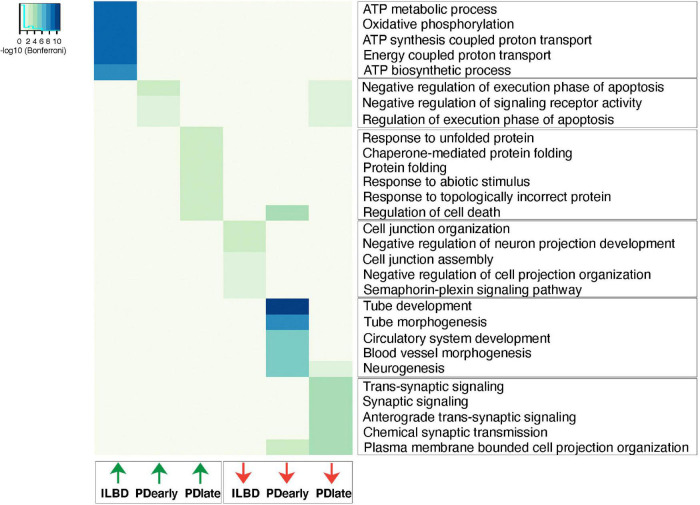
Up- and down-regulated biological processes in PDearly, PDlate and incidental Lewy body disease. Top significant biological process categories from differentially expressed genes (padj < 0.05, RPKM in controls > 1, fold change > 1.5) as analyzed by ToppGene suite. Heatmap indicates significance of each individual GO-term as revealed by Bonferroni test.

Gene ontology analysis was performed to identify pathways that are enriched among genes that are differentially expressed at higher levels in PD and ILBD samples (padj < 0.05, RPKM in controls > 1, fold change > 1.5; Figure 6 and Supplementary Table 5). Terms related to negative regulation of apoptosis appears to be upregulated in PDearly samples. However, it should be noted that almost all genes contributing to these GO-terms belong to the same family of genes with homology to the mitochondrial encoded *MT-RNR2* (humanin) gene (Supplementary Figure 6). Humanin has been shown to have anti-apoptotic and neuroprotective effects and has been associated with Alzheimer’s disease (Hashimoto et al., 2001). Several GO-terms relating to regulation of apoptosis and programmed cell death were also identified when analyzing enriched genes in PDlate (Figure 6 and Supplementary Table 5). Another set of GO terms among genes enriched in late PDlate samples were dominated by terms related to the response to unfolded proteins and chaperone-mediated protein folding. Genes associated with the endoplasmic reticulum (ER) unfolded protein response pathways (UPR) are included among these genes, e.g., *XBP1*, *CHAC1*, and *DDIT3* (*CHOP*), but also many other genes encoding proteins that regulate cytoplasmic response to unfolded proteins (Supplementary Figure 6 and Supplementary Table 5). Enriched pathways identified from analysis of downregulated genes demonstrated that there was a significant downregulation of synaptic signaling and neuronal projection organization in PDlate samples suggesting defects in synaptic transmission and axon/dendrite integrity, which are hallmarks of PD pathology (Supplementary Figure 7). Finally, in ILBD samples, downregulated genes were associated with GO terms relating to genes involved in cell junction. Strikingly, enriched genes were strongly linked to terms associated with oxidative phosphorylation (Figure 6 and Supplementary Table 5).

## Discussion

Understanding changes in gene expression in PD is important for several reasons. Identifying potentially dysregulated genes in PD can provide new biomarkers, information on pathological mechanisms, and potential novel therapeutic targets. Here we analyzed gene expression changes in *post-mortem* tissue samples from PD patients early after diagnoses; PD patients at advanced stages of disease; and ILBD patients, respectively. Our goal was to analyze gene expression specifically in mDA neurons of the SNc and for this reason LCM was used to isolate NM+ cells from frozen tissue sections dissected from substantia nigra. The comprehensive analysis using RNAseq combined with LCM isolated cells has given novel insights into how gene expression changes during PD progression.

Several previous studies have analyzed changes in gene expression in *post-mortem* brain tissue from PD patients (Cantuti-Castelvetri et al., 2007; Bossers et al., 2009; Simunovic et al., 2009; Zheng et al., 2010; Elstner et al., 2011; Riley et al., 2014). A relatively modest concordance is seen across different studies reflecting the challenges when analyzing differences in gene expression in patient samples (Oerton and Bender, 2017). Several sources of variation are evident: (*i)* patient samples differ due to varying disease progression and clinical features of disease; (*ii)* differences in tissue quality, e.g., due to different PMIs is an additional source of variability; (*iii)* precision in dissection is challenging and unavoidably introduces substantial differences between studies; (*iv)* dissected tissue from substantia nigra is complex with regard to cellular composition and includes different neuronal and non-neuronal cell types. Of note, mDA neurons only comprise a minority of dissected cells and such cellular diversity further complicates interpretation of data, in particular when mRNA has been extracted from homogenized tissue samples consisting of mixed ventral midbrain cell types; (*v)* the method used for detecting gene expression varies between studies (hybridization arrays or RNAseq). Most importantly, different commercial hybridization platforms are well known to differ with regard to sensitivity and specificity in gene detection.

Our goal has been to increase the precision in analysis by using LCM of NM+ mDA neurons to limit the variability in gene expression resulting from inclusion of surrounding non-DA neurons that may be more or less influenced by the disease process. In addition, quantifying gene expression by RNAseq has a superior sensitivity and dynamic range compared to hybridization arrays, which have been used in most previous studies. Moreover, the tissue samples used here are of high quality with relatively short PMI and have been annotated with regard to clinical features and disease progression (Supplementary Figure 1). Finally, our study included samples originating both from patients early after diagnosis and from more advanced stages of disease. The inclusion of tissue from early diagnosed PD patients allowed us to highlight the important question of how disease duration influences gene expression. Finally, the analysis of ILBD samples provided an interesting complement to PD and control samples.

Our analysis showed that differentially expressed genes showed only a modest overlap when comparing changes in PDearly and PDlate samples, thus suggesting relatively clear differences in remaining NM+ mDA neurons dependent on disease duration. To understand the reason for these differences it is important to consider the rate of cell loss in PD. In a recent study, mDA neurons cell loss was studied in post-diagnostic intervals from 1 to 27 years (Kordower et al., 2013). Importantly, a rapid (50–90%) loss of NM+ neurons was observed in the SNc in the first few years following diagnosis while only minimal additional loss is seen at later stages. Based on these histological data it can be concluded that the vast majority of remaining mDA neurons in PDlate samples are stably surviving NM+ mDA neurons since only minimal additional neuronal loss is seen at later stages (Kordower et al., 2013). In contrast, at least a proportion of cells analyzed in PDearly samples are likely more acutely influenced by pathological stress as they will eventually degenerate. It seems likely that these differences are the basis for some of the substantial differences in gene expression observed in the two categories of PD samples. Accordingly, we hypothesized that most of the cells in PDlate samples are likely stably maintained in these PD patients and that gene expression in late PDlate samples, at least in part, can be explained by a progressive selection of cells that are more robust and with a higher propensity to survive over time in PD. Notably, in PDlate cases a majority of markers of human VTA mDA neurons were found to be enriched while a majority of the human SNc markers were downregulated (Figure 5). This implies that mDA neurons with a subtype profile that is more similar to neurons of the VTA may represent a less vulnerable subpopulation that survives in SN of PD patients and for this reason contributes to the gene enrichment in samples isolated from late stage PD samples. Presumably, such VTA or “VTA-like” cells are normally localized in the more resilient dorsal tier of the SNc or in lateral parts of the VTA (Fearnley and Lees, 1991). In contrast, it seems likely that differential gene expression in early samples, at least to some extent, reflects more immediate responses to cellular stress in the more vulnerable ventral tier of the SNc. Finally, it should be noted that the early post-diagnosis cases hade on average a relatively high age of disease onset (83 years) as compared to late post-diagnosis patients (68 years). This most likely reflects that death rarely occurs early after onset in younger patients and those samples are therefore uncommon in the tissue collection. However, we cannot rule out that these differences have also contributed to some of the differences in gene expression profiles seen between the two groups.

In addition to the analyses of gene expression in mDA neuron samples isolated after short and long disease duration, respectively, our study also considered how gene expression in samples from individuals identified with ILBD related to PD and control samples. It remains unclear if ILBD represents a pre-clinical stage of PD or if it represents a unique pathological entity that does not cause PD symptoms (Adler et al., 2010). It could be reasoned that a similar profile of up- or downregulated genes with those seen in PD < 4 years samples would be seen if ILBD represents a pre-clinical phase of PD. However, as ILBD has nigrostriatal dopaminergic losses midway between normal and PD, it seems a reasonable candidate for a preclinical stage and the differences between gene expression profiles seen in ILBD, PDearly and PDlate subjects may thus reflect distinct stages of one and the same disease (Beach et al., 2008; DelleDonne et al., 2008; Dickson et al., 2008; Iacono et al., 2015). If this is the case it is tempting to speculate that the striking upregulation of oxidative phosphorylation pathways seen in ILBD (Figure 6) may indicate a neuroprotective response at early stages of disease development.

Taken together, the results presented in this study provides a unique view of gene expression seen in remaining mDA neurons in patients with PD at different stages of disease duration in comparison to age-matched controls and ILBD cases. Further functional studies of identified regulated genes will be required to address their potential roles in relation to neuron vulnerability or neuroprotection.

## Data Availability Statement

The datasets generated and analyzed for this study can be found in the Gene Expression Omnibus, National Center for Biotechnology Information, using accession number GSE182622.

## Ethics Statement

This study involving human participants was reviewed and approved by the Western Institutional Review Committee (WIRB), Seattle, Washington. The patients/participants provided their written informed consent to participate in this study.

## Author Contributions

TP and KT: conceptualization. KT, NV, LG, LD, and HL-M: formal analysis and investigation. TB, GS, and CA: resources. TP, KT, and NV: writing manuscript. KT and NV: computational analysis. TP: supervision. All authors contributed to the article and approved the submitted version.

## Conflict of Interest

The authors declare that the research was conducted in the absence of any commercial or financial relationships that could be construed as a potential conflict of interest.

## Publisher’s Note

All claims expressed in this article are solely those of the authors and do not necessarily represent those of their affiliated organizations, or those of the publisher, the editors and the reviewers. Any product that may be evaluated in this article, or claim that may be made by its manufacturer, is not guaranteed or endorsed by the publisher.

## Footnotes

1
https://www.bannerhealth.com/services/research/locations/sun-health-institute/programs/body-donation/tissue

## Abbreviations

ER: endoplasmic reticulum
GO: gene ontology
ILBD: incidental Lewy body disease
LCM: laser-capture microdissection
mDA: midbrain dopamine
NM: neuromelanin
NM+: neuromelanin-positive
PCA: principal component analysis
PC2: second principal component
PD: Parkinson’s disease
PDearly: Parkinson’s disease duration of 2–4 years
PDlate: Parkinson’s disease duration of 5–24 years
PMI: post-mortem interval
RNAseq: RNA sequencing
RPKM: reads per million mapped reads
SNc: substantia nigra pars compacta
UPR: unfolded protein response
VTA: ventral tegmental area.
