# Supplementary material for: Disease Duration Influences Gene Expression in Neuromelanin-Positive Cells From Parkinson’s Disease Patients
Source: Front Mol Neurosci. 2021 Nov 11;14:763777. doi: 10.3389/fnmol.2021.763777 (PMC8632647; doi:10.3389/fnmol.2021.763777)
Supplement: Supplementary file 6 [file Data_Sheet_1.docx]

Supplementary Material

1. **Supplementary Figures and Tables**
   1. **Captions for Supplementary Figures**

**Figure S1 Cases included in the study.** Control, Parkinson’s disease, and incidental Lewy body disease cases used in the study. Indicated is expired age; gender (M=male, F=female); postmortem index in hours (PMI); Braak-score; disease duration (years) and number of samples generated for RNAseq from each patient (Samples).

**Figure S2 Validation of neuromelanin positive samples. (A)** Spearman correlations of NM+ samples. **(B)** Expression level of markers indicative of astrocytes (*AQP4* and *GFAP*); oligodendrocytes (*OLIG1* and *OLIG2*) and vascular fibroblast-like cells (*COL1A1* and *COL1A2*) in log_2_(RPKM+1). **(C)** Top significant biological process categories from differentially expressed genes between NM+ and cortex samples (padj <0.05, RPKM in controls >1, fold change >1.5) as analyzed by ToppGene suite. Heatmap indicates significance of each individual GO-term as revealed by Bonferroni test.

**Figure S3 Extended analysis of segregation of PD samples. (A)** Two dimensional PCA of Control, Parkinson’s disease and incidental Lewy body disease samples. PC5 separates Parkinson’s disease from Control and incidental Lewy body disease samples**.** Samples are color-coded by disease. **(B, C)** Two dimensional PCA of Control and Parkinson’s disease samples. PC5 separates Parkinson’s disease from Control samples**.** Samples are color-coded by disease and disease duration. **(D)** Two dimensional PCA of PDlate and PDearly samples. PC2 separates PDlate from PDearly samples**.** Samples are color-coded by disease duration. **(E)** Two dimensional PCA (PC2, PC3) of PD samples. Samples are color-coded to indicate gender, PMI, BRAAK score and expired age.

**Figure S4 Gene expressions overlap and changes. (A)** Overlap of upregulated and downregulated genes between PDearly, PDlate and ILBD. **(B)** Heatmap visualizing expression of top-upregulated genes in incidental Lewy body disease.

**Figure S5 Top downregulated genes in in PDearly, PDlate and incidental Lewy body disease.** Heatmap visualizing expression of top-downregulated genes in PDearly, PDlate and incidental Lewy body disease. Color-coding indicates sample types as indicated.

**Figure S6 Genes related to upregulated biological processes in PDearly, PDlate and incidental Lewy body disease.** Heatmap of enriched genes from top upregulated significant biological process categories in PDearly, PDlate and incidental Lewy body disease.

**Figure S7 Genes related to downregulated biological processes in PDearly, PDlate and ILBD.** Heatmap of enriched genes from top downregulated significant biological process categories in PDearly, PDlate and incidental Lewy body disease.

- 1. **Captions for Supplementary Tables**

**Table S1** Gene list from DESeq2 analysis of NM+ samples vs Cortex. DESeq2 outcome: baseMean = average of the normalized count values, dividing by size factors, taken over all samples; log2FoldChange= effect size estimate; lfcSE= standard error of the log2FoldChange estimate; stat= Wald statistic; pvalue= Wald test *p*-value; padj= Benjamini-Hochberg adjusted *p*-value corrected for multiple testing.

**Table S2** Gene list from DESeq2 analysis of Control vs PDearly samples (disease duration 2-4 years). DESeq2 outcome: baseMean = average of the normalized count values, dividing by size factors, taken over all samples; log2FoldChange= effect size estimate; lfcSE= standard error of the log2FoldChange estimate; stat= Wald statistic; pvalue= Wald test *p*-value; padj= Benjamini-Hochberg adjusted *p*-value corrected for multiple testing.

**Table S3** Gene list from DESeq2 analysis of Control vs PDlate samples (disease duration 5-24 years). DESeq2 outcome: baseMean = average of the normalized count values, dividing by size factors, taken over all samples; log2FoldChange= effect size estimate; lfcSE= standard error of the log2FoldChange estimate; stat= Wald statistic; pvalue= Wald test *p*-value; padj= Benjamini-Hochberg adjusted *p*-value corrected for multiple testing.

**Table S4** Gene list from DESeq2 analysis of Control vs incidental Lewy body disease samples. DESeq2 outcome: baseMean = average of the normalized count values, dividing by size factors, taken over all samples; log2FoldChange= effect size estimate; lfcSE= standard error of the log2FoldChange estimate; stat= Wald statistic; pvalue= Wald test *p*-value; padj= Benjamini-Hochberg adjusted *p*-value corrected for multiple testing.

**Table S5** List of significantly up- and down-regulated biological process categories and their related genes for PDearly, PDlate and incidental Lewy body disease samples as analyzed by ToppGene suite.
