# Supplementary figures and images for: Disease Duration Influences Gene Expression in Neuromelanin-Positive Cells From Parkinson’s Disease Patients
Source: Front Mol Neurosci. 2021 Nov 11;14:763777. doi: 10.3389/fnmol.2021.763777 (PMC8632647; doi:10.3389/fnmol.2021.763777)

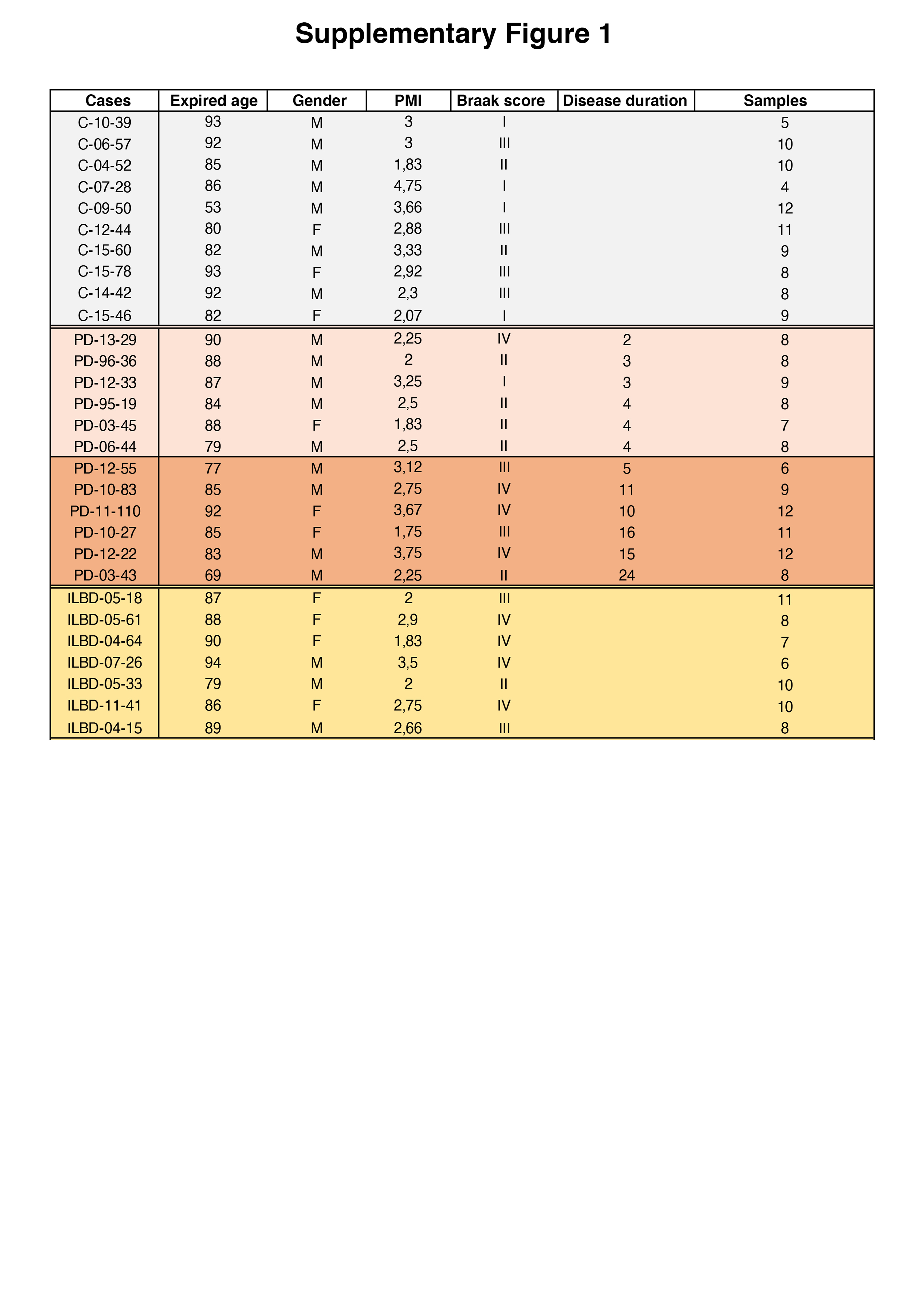

Supplement: Supplementary file 7 [file Image_1.JPEG]

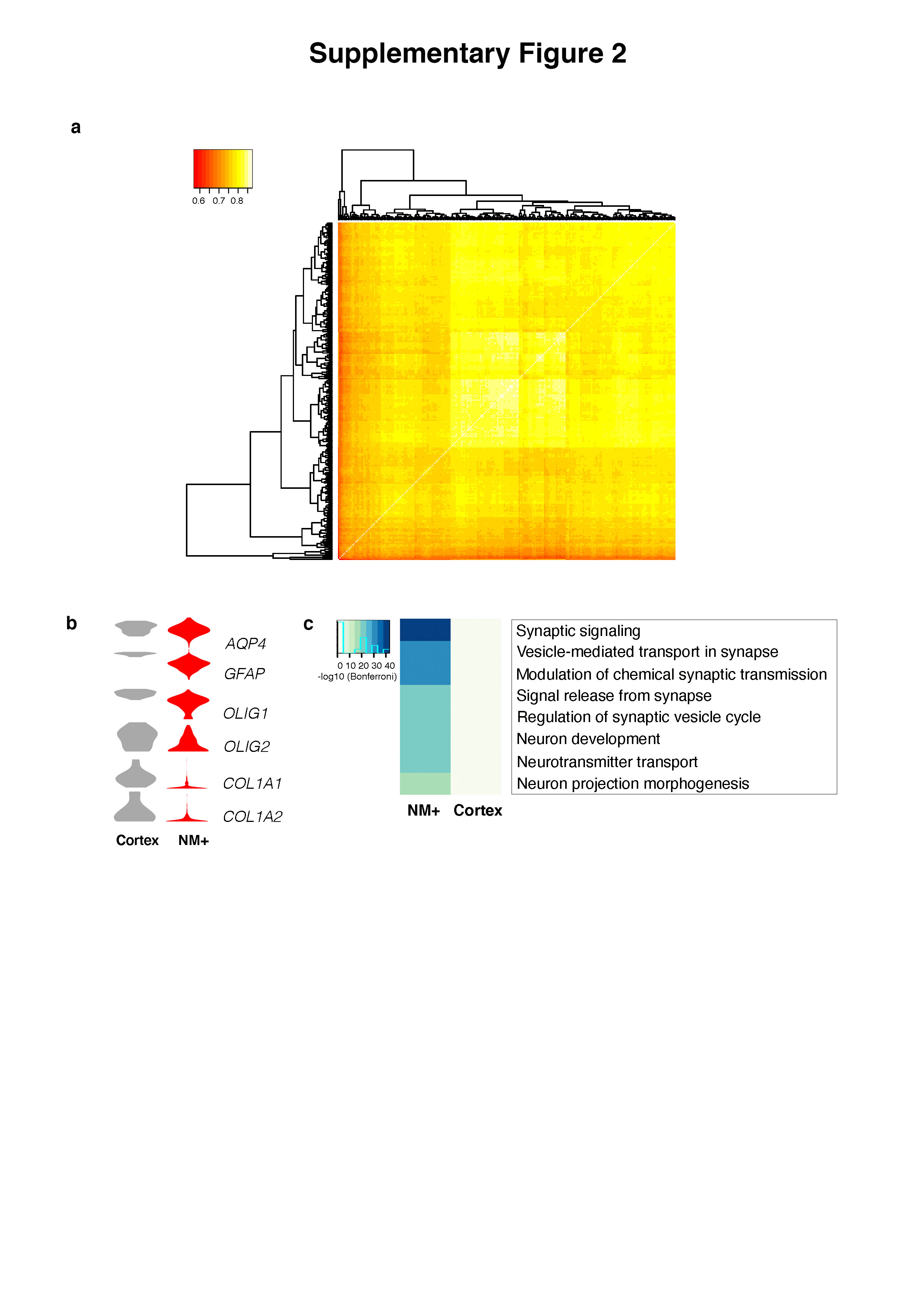

Supplement: Supplementary file 8 [file Image_2.JPEG]

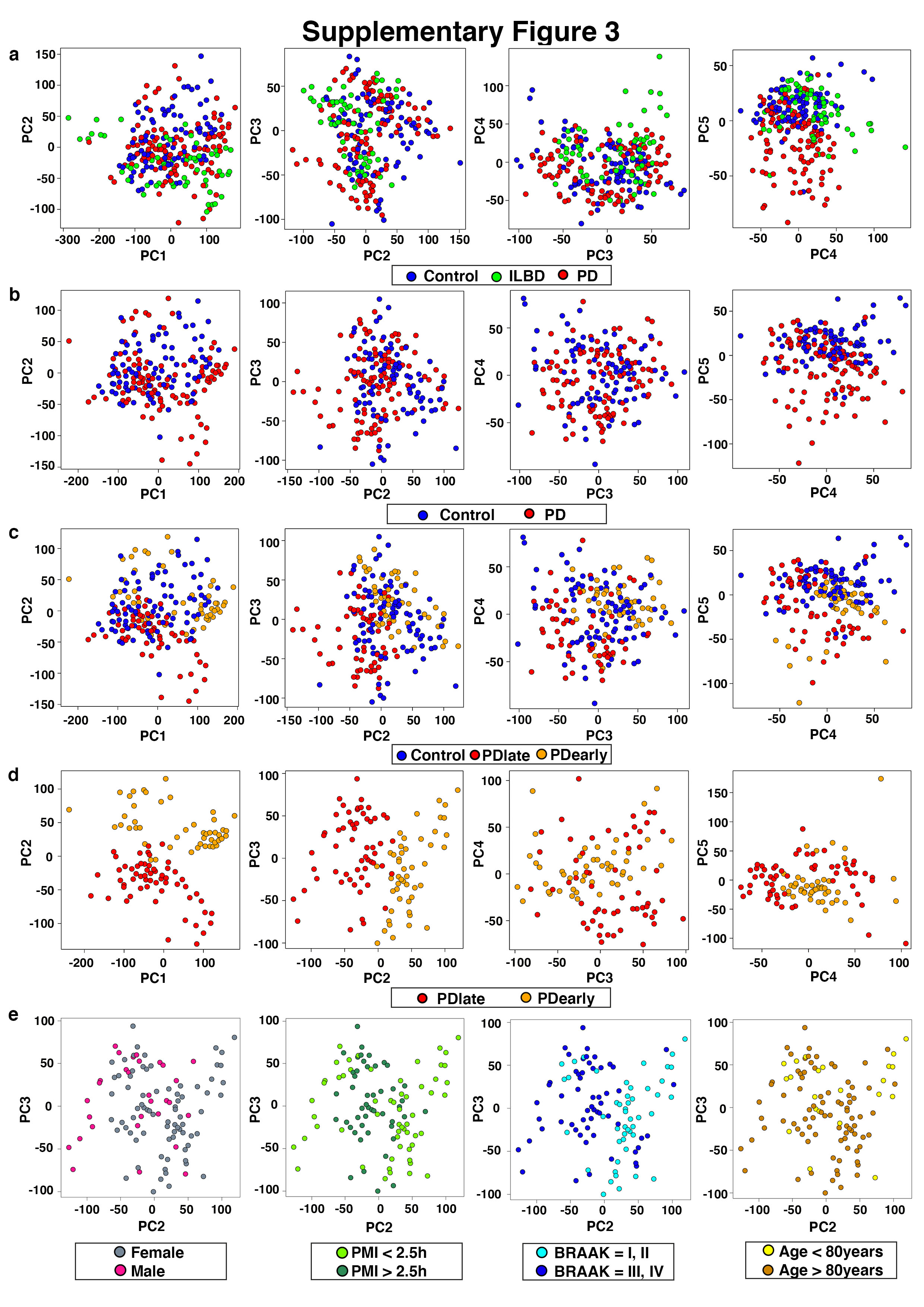

Supplement: Supplementary file 9 [file Image_3.JPEG]

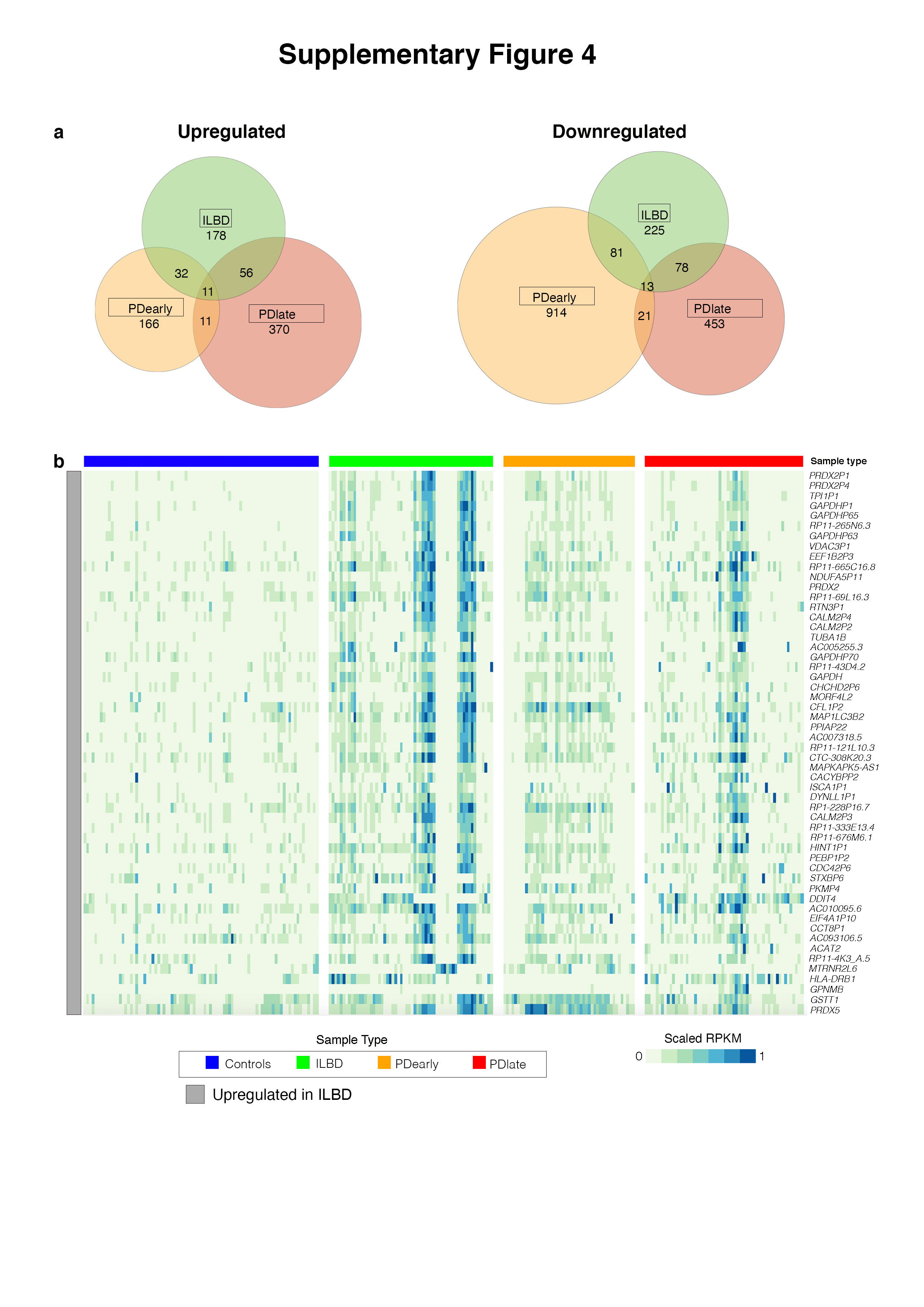

Supplement: Supplementary file 10 [file Image_4.JPEG]

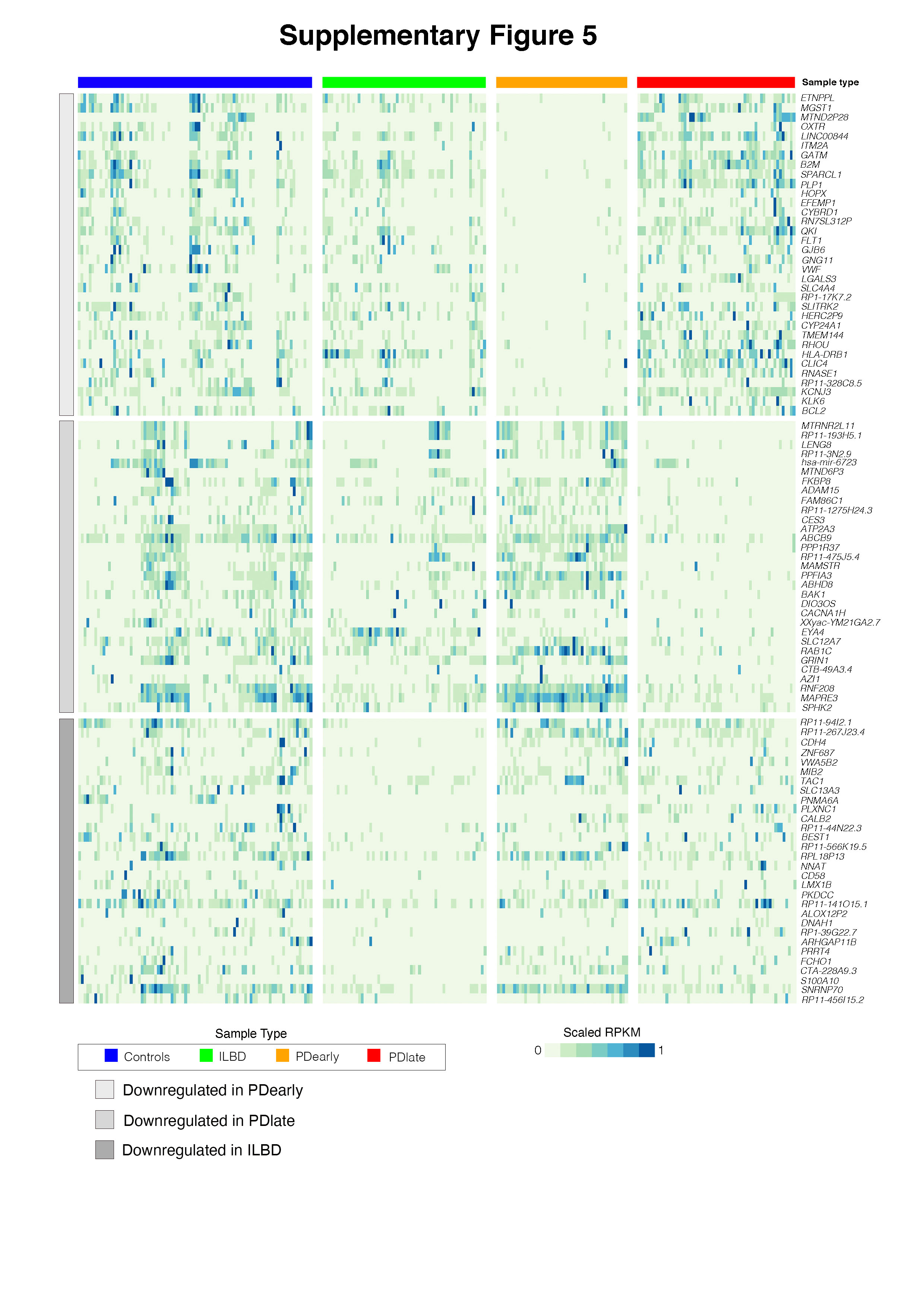

Supplement: Supplementary file 11 [file Image_5.JPEG]

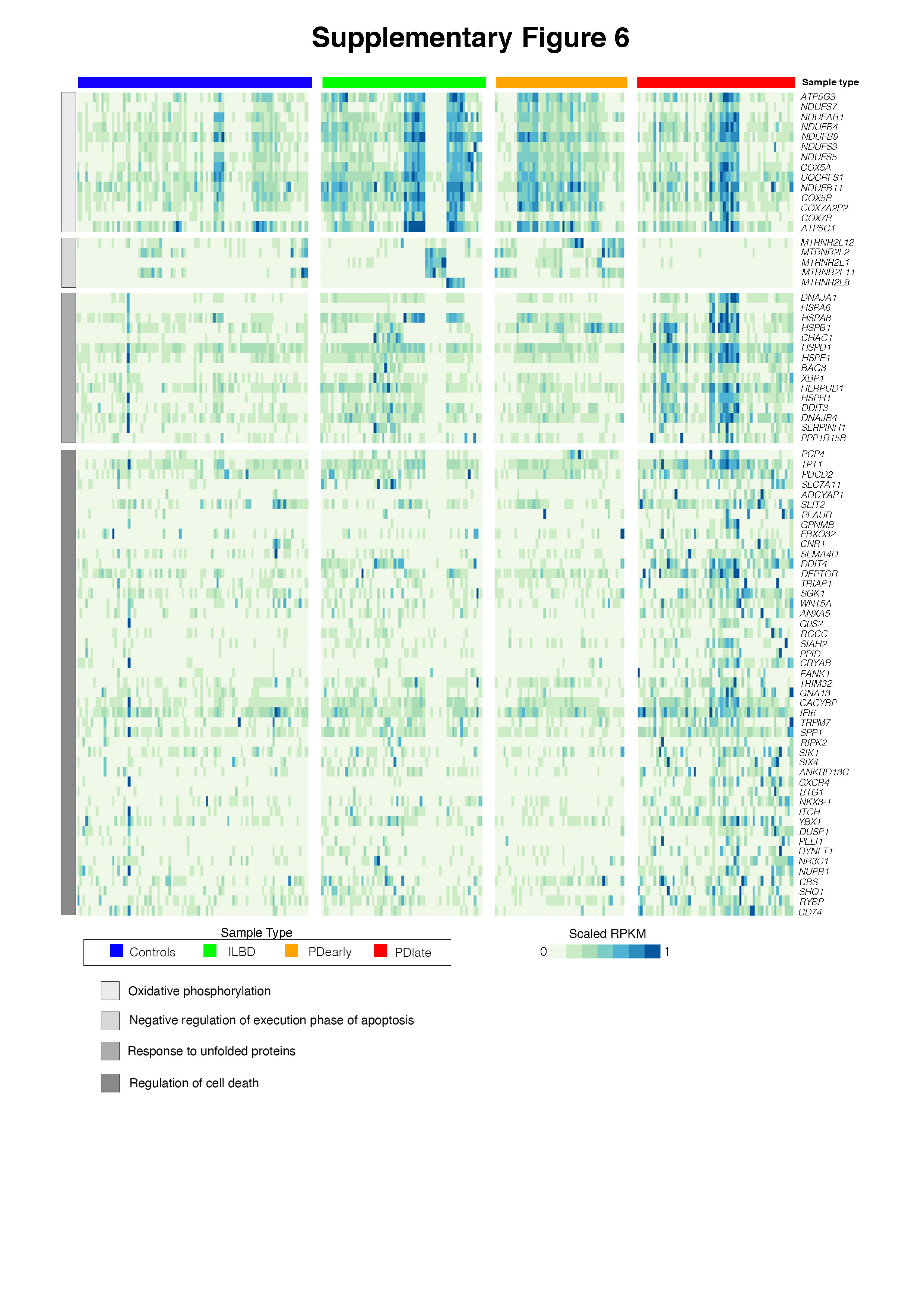

Supplement: Supplementary file 12 [file Image_6.JPEG]

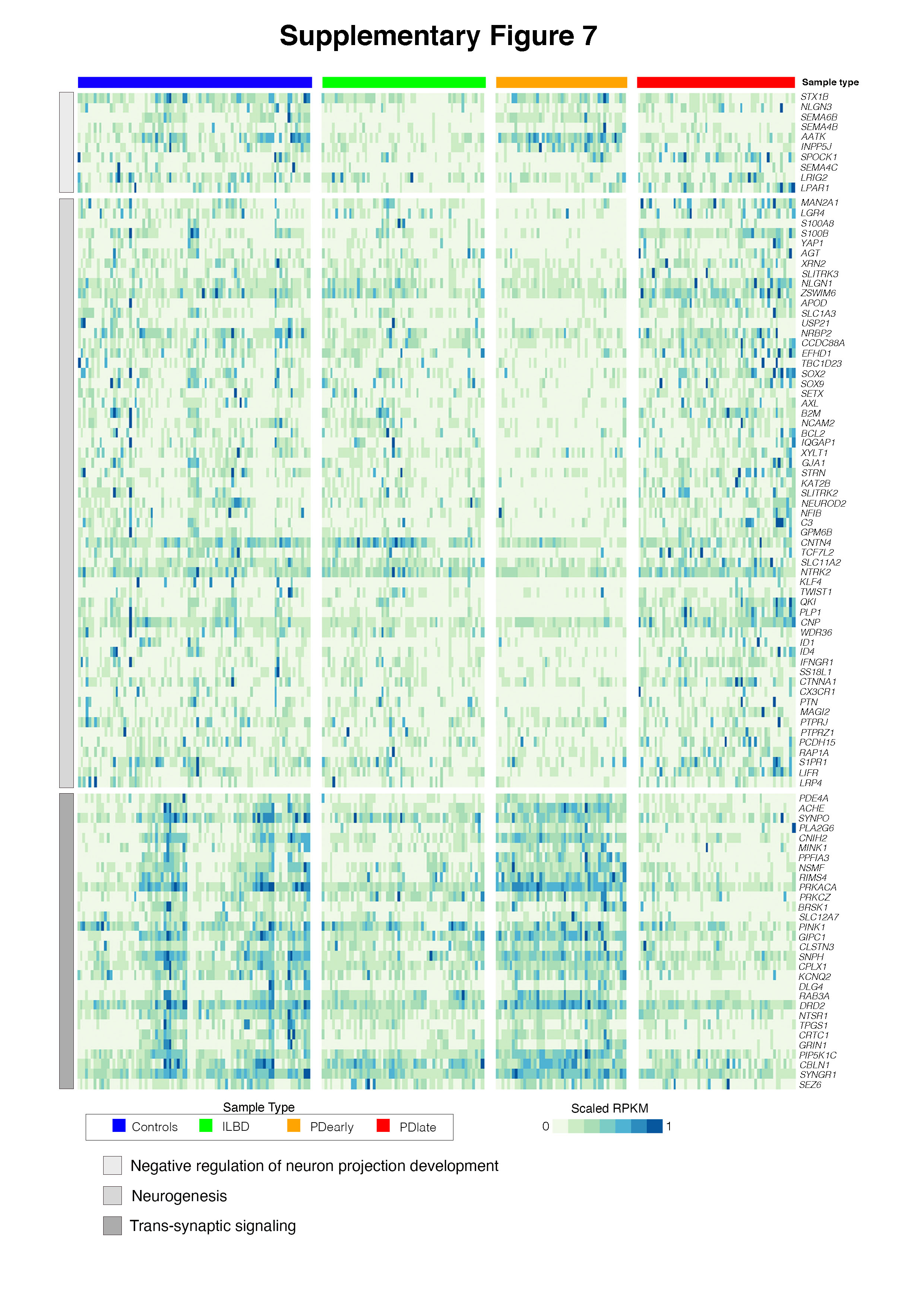

Supplement: Supplementary file 13 [file Image_7.JPEG]
